# Supplementary material for: Coherent exciton-vibrational dynamics and energy transfer in conjugated organics
Source: Nat Commun. 2018 Jun 13;9:2316. doi: 10.1038/s41467-018-04694-8 (PMC5998141; doi:10.1038/s41467-018-04694-8)
Supplement: Supplementary file 1 — Supplementary Information [file 41467_2018_4694_MOESM1_ESM.pdf]

Supplementary Information

Coherent Exciton-Vibrational Dynamics and Energy Transfer in Conjugated Organics

Nelson et al.

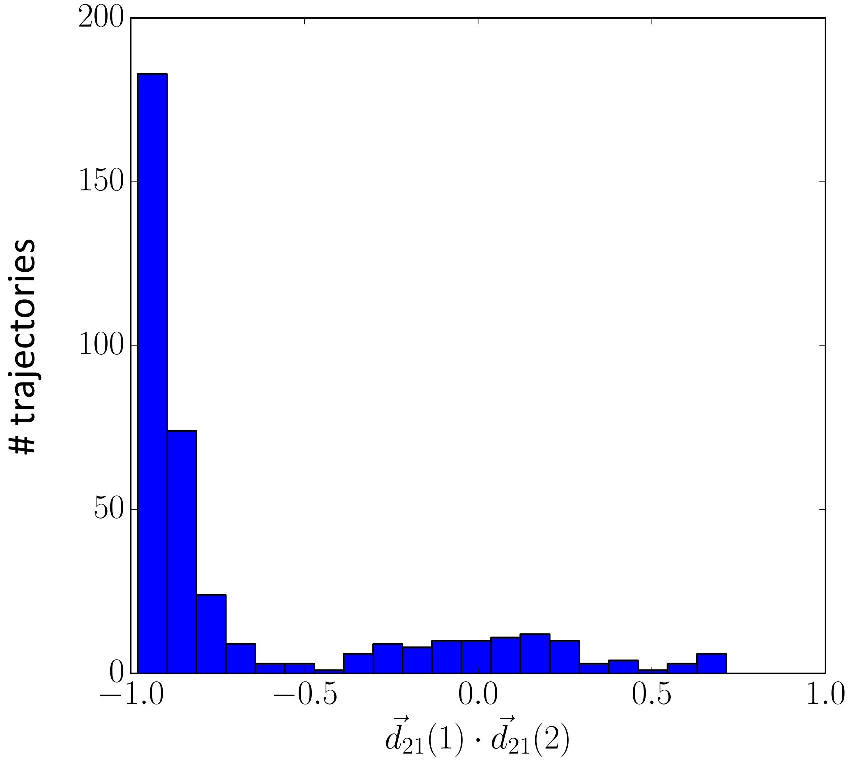

Supplementary Figure 1. The overlap between the NAC vector fractions for the DTA dimer. Here the monomers are translated to their respective centers of mass and rotated according to their inertial axes so that both monomers superimpose. The fraction of NAC vector corresponding to each monomer is also rotated and renormalized. The overlap between the two reoriented NAC vector fractions is calculated and used to construct the histogram for all trajectories. Most values are close to -1 indicating the dephasing of one half of NAC vector related to the other in all trajectories. The monomers move asymmetrically leading to the wave behavior in the electronic transition density localization.

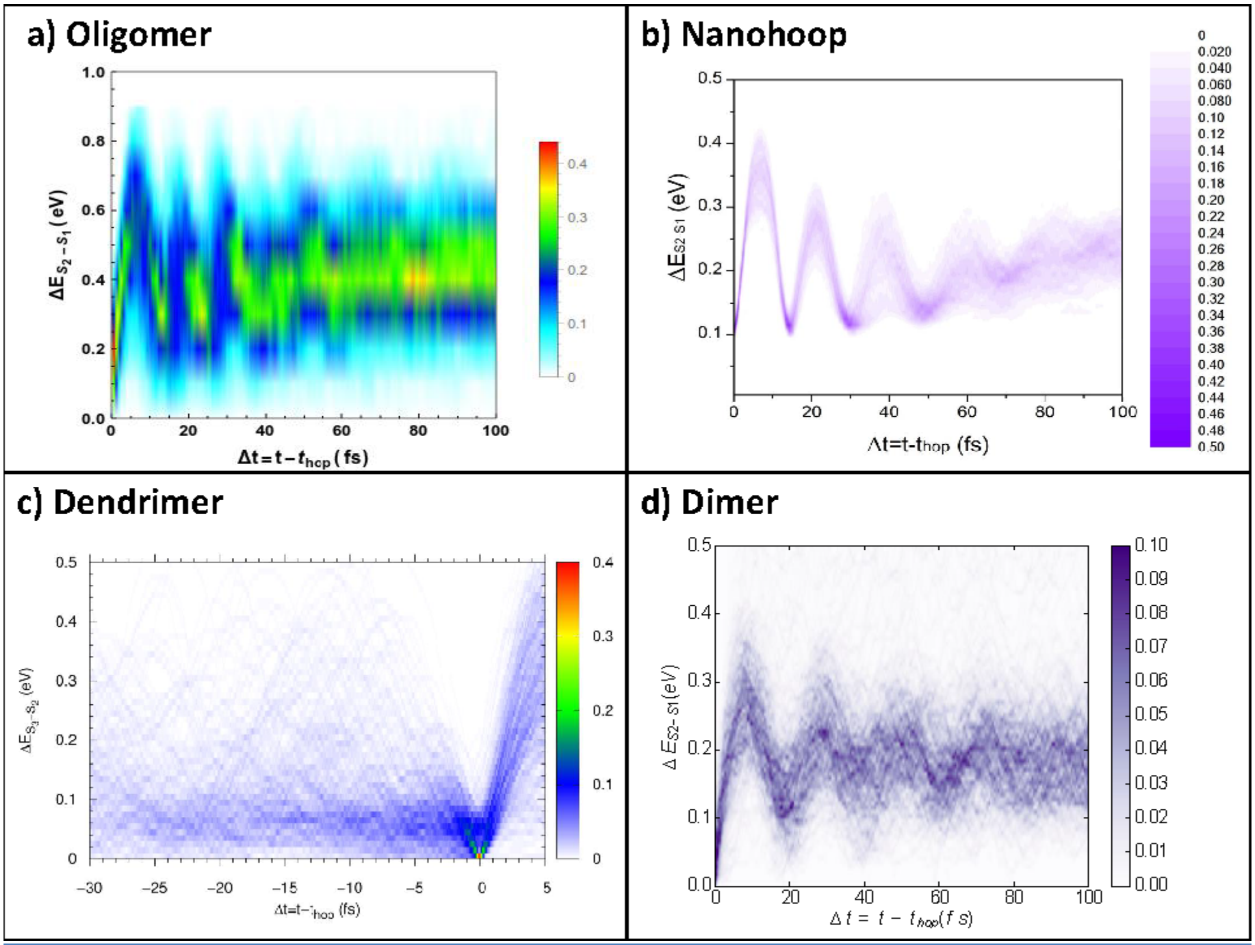

Supplementary Figure 2. Plots of the distribution of S_2-S_1 (S_3-S_2 for dendrimer) energy gaps after the effective hops. For the dendrimer (**c**), nanohoop (**b**) and dimer (**d**), at the moment of the non-adiabatic transition (Δt=0), the energy gap is small and the distribution is very narrow (i.e., all trajectories exhibit a small energy gap). The average values of the energy gaps are $\Delta E(S_3-S_2) \sim 0.04$ eV, $\Delta E(S_2-S_1)\sim 0.11$ eV, and $\Delta E(S_2-S_1)\sim 0.06$ eV for **c**), **b**) and **d**), respectively. The regions where the electronic states are not well separated require non-adiabatic treatment. In contrast, the corresponding gap for the oligomer (**a**) is $\Delta E(S_2-S_1)\sim$ 0.22 eV, and the dynamics after crossing to the S_1 state can be denoted as essentially adiabatic.

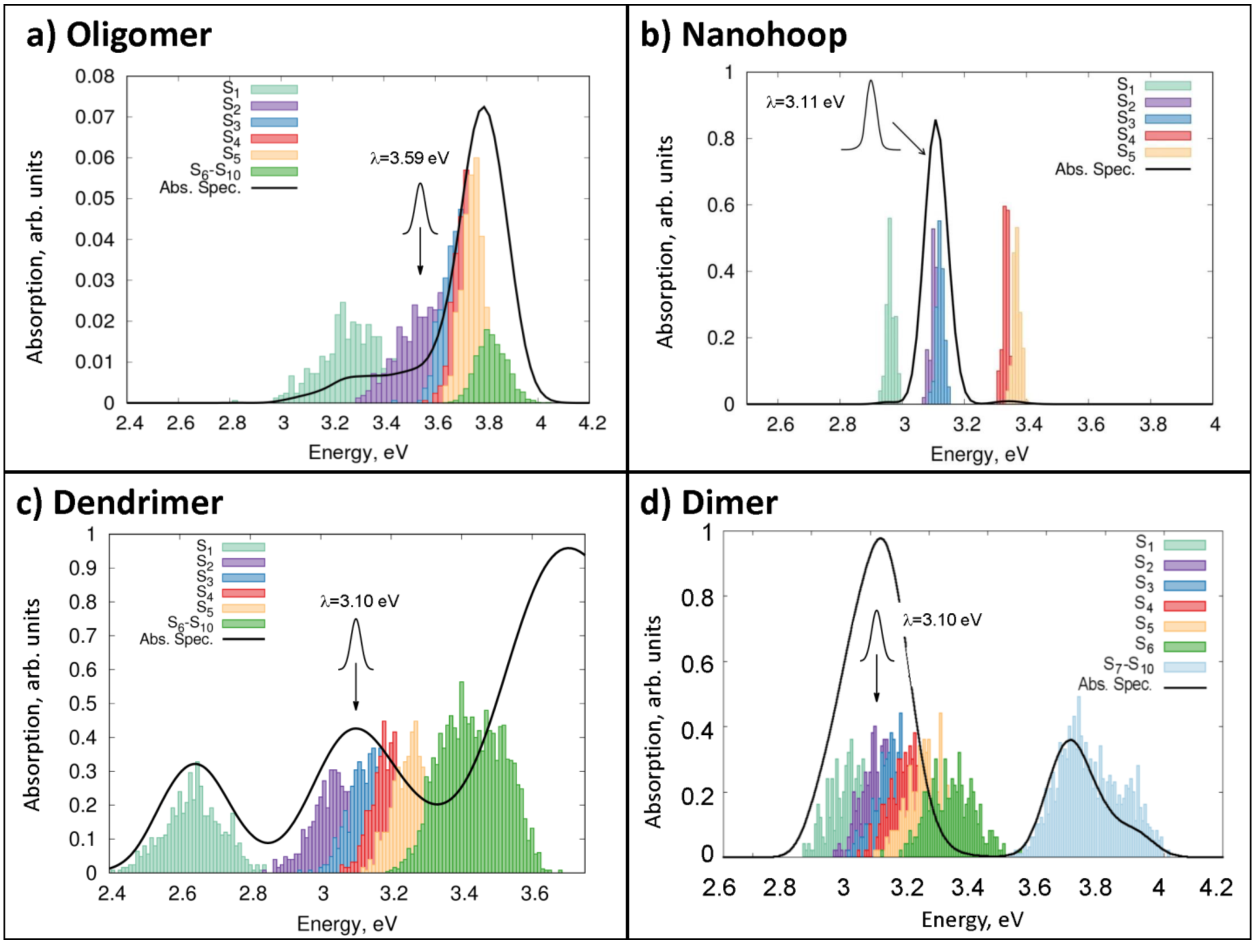

Supplementary Figure 3. Equilibrated absorption spectra and density of excited states (DOES). The plots are for oligomer (**a**), nanohoop (**b**), dendrimer (**c**) and dimer (**d**) model systems calculated at room temperature (300K). The spectra show the contributions of individual excited states, plotted as a distribution over the equilibrated ensemble, and the excitation wavelength. For each trajectory, only a single initial state is populated. Among the ensemble, the initial state may vary due to conformational disorder.

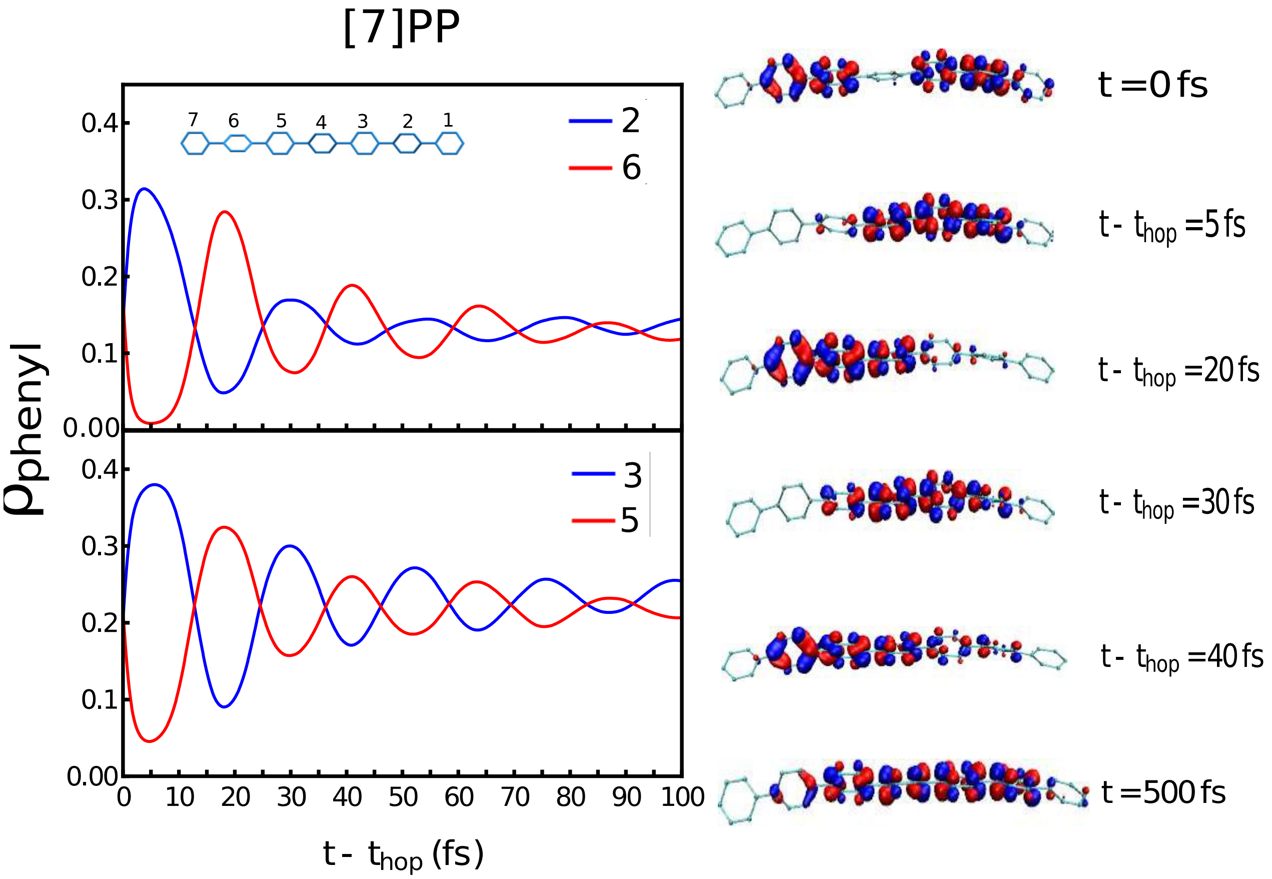

Supplementary Figure 4. The evolution of the fraction of transition density. Plotted is the evolution of the fraction of transition density localized on rings 2/6 (top panel) and 3/5 (bottom panel) of the 7-polyphenylene oligomer and corresponding snapshots of the electronic transition density during NEXMD simulations of photoexcited dynamics. The sloshing of electronic transition density between left and right halves of the oligomer can be seen from the out-of-phase oscillations in the transition density plots.

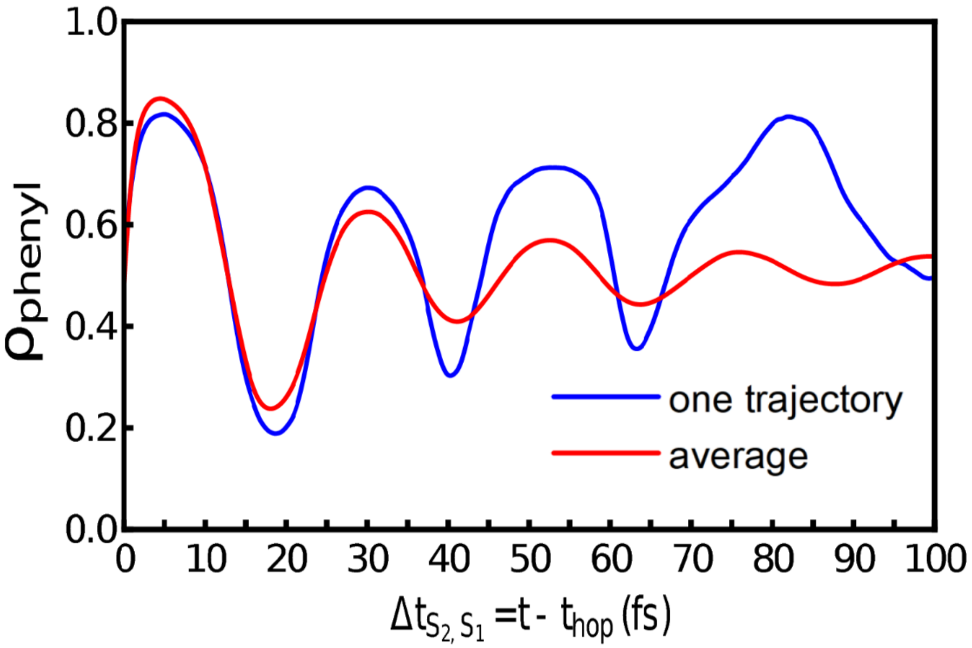

Supplementary Figure 5. The evolution of the fraction of transition density localized on one half of the oligomer. The results are obtained with NEXMD simulations of photoexcited dynamics for a single trajectory and for the average ensemble of trajectories. Both plots confirm the oscillations of transition density between the two halves of the oligomer induced by the hop from S_2 to S_1.

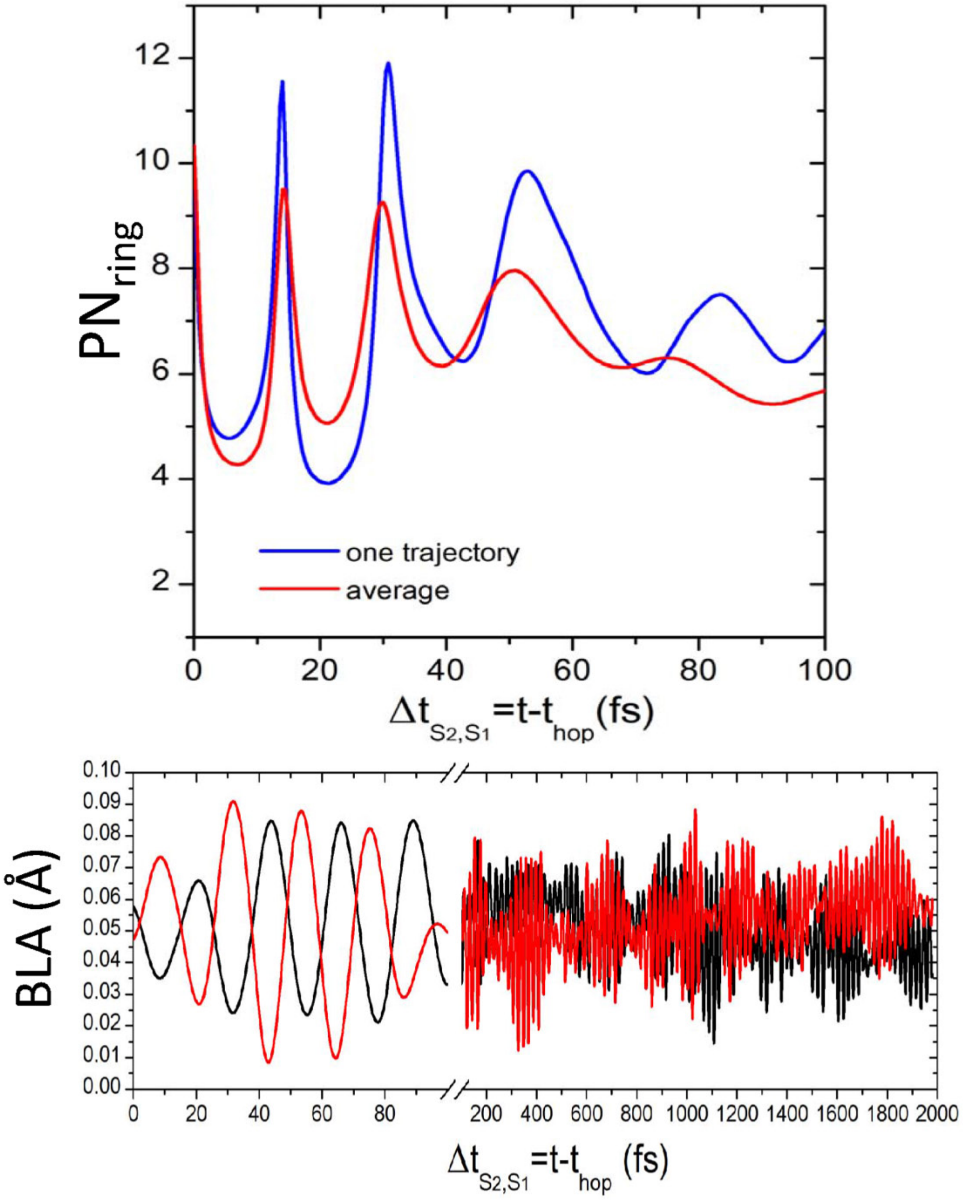

Supplementary Figure 6. The evolution of the participation number (PN) and bond-length alternation (BLA) in the nanohoop. The results are obtained with NEXMD simulations of the photoexcited dynamics at the time of the hop from S_2 to S_1. The participation number is defined with each ring as a unit and shows oscillations between excitation localized over 4-6 rings (PN=4-6) corresponding to half of the nanohoop and excitations delocalized over nearly the entire nanohoop (PN=8-12). The behavior observed in the single trajectory, is also true for the average ensemble of trajectories. The BLA directly after the hop is plotted for the two halves of the nanohoop (black and red curves) and show alternating oscillations with respect to the molecular halves indicating the change in conjugation length caused by the excitation localization on different halves of the nanohoop. The oscillations decay within 200fs after the non-adiabatic transition.

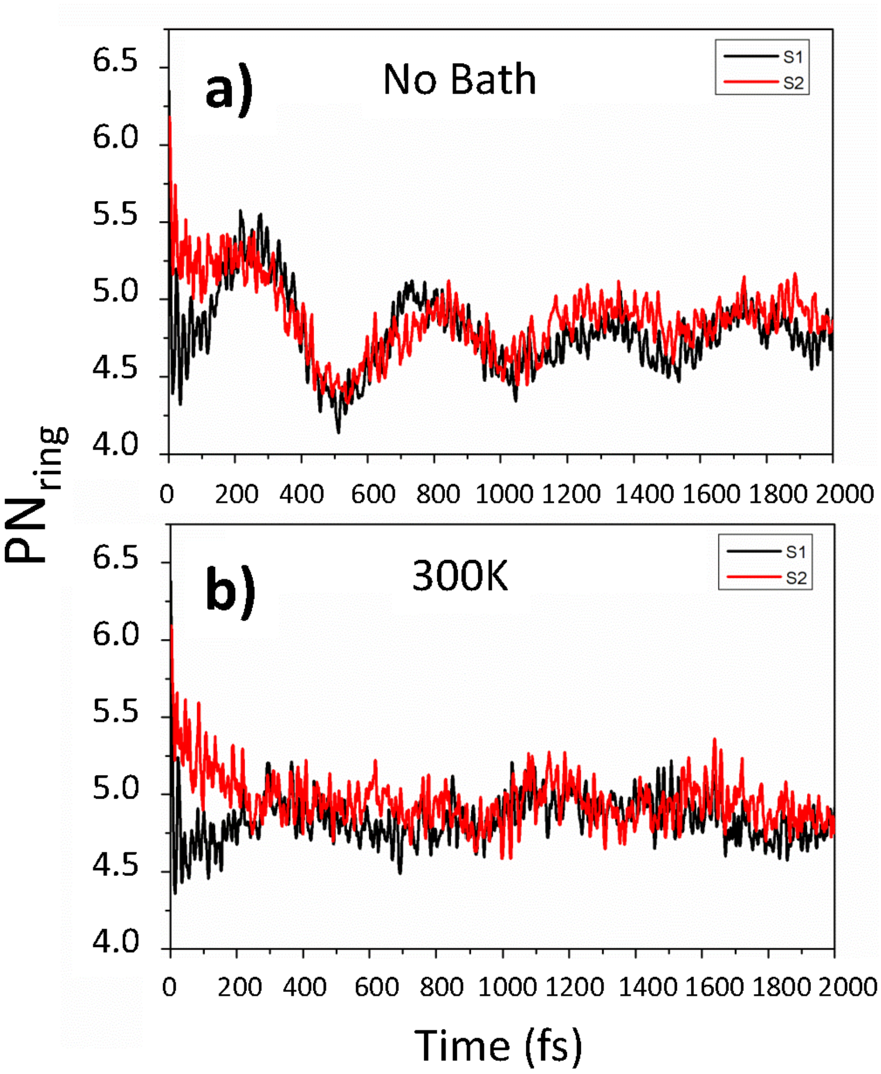

Supplementary Figure 7. The average participation number (PN) for the nanohoop. The results are obtained with NEXMD simulations of the photoexcited dynamics without and with the Langevin bath. In the absence of the bath (**a**), the participation number for dynamics on the S_1 (black) and S_2 (red) potential energy surfaces both experience oscillations between localized and delocalized states. These oscillations are damped in the presence of the thermal bath (**b**).
