# Supplementary material for: Coherent exciton-vibrational dynamics and energy transfer in conjugated organics
Source: Nat Commun. 2018 Jun 13;9:2316. doi: 10.1038/s41467-018-04694-8 (PMC5998141; doi:10.1038/s41467-018-04694-8)
Supplement: Supplementary file 4 — Description of Additional Supplementary Files [file 41467_2018_4694_MOESM4_ESM.pdf]

Description of Additional Supplementary Files

File Name: Supplementary Movie 1
Description: The movie shows evolution of transition density in the DTA dimer (left panel) and concurrent time-dependent changes of the fraction of transition density on a single monomer (right panel) along a single trajectory (blue curve) and averaged across trajectory ensemble (red curve) calculated using the NEXMD code.
